# Post-transcriptional Mechanisms Contribute Little to Phenotypic Variation in Snake Venoms

**DOI:** 10.1534/g3.115.020578

**Published:** 2015-09-09

**Authors:** Darin R. Rokyta, Mark J. Margres, Kate Calvin

**Affiliations:** * Department of Biological Science, Florida State University, Tallahassee, Florida 32306; † College of Medicine, Florida State University, Tallahassee, Florida 32306

**Keywords:** genotype–phenotype map, transcriptome, proteome, venom, snake

## Abstract

Protein expression is a major link in the genotype–phenotype relationship, and processes affecting protein abundances, such as rates of transcription and translation, could contribute to phenotypic evolution if they generate heritable variation. Recent work has suggested that mRNA abundances do not accurately predict final protein abundances, which would imply that post-transcriptional regulatory processes contribute significantly to phenotypes. Post-transcriptional processes also appear to buffer changes in transcriptional patterns as species diverge, suggesting that the transcriptional changes have little or no effect on the phenotypes undergoing study. We tested for concordance between mRNA and protein expression levels in snake venoms by means of mRNA-seq and quantitative mass spectrometry for 11 snakes representing 10 species, six genera, and three families. In contrast to most previous work, we found high correlations between venom gland transcriptomes and venom proteomes for 10 of our 11 comparisons. We tested for protein-level buffering of transcriptional changes during species divergence by comparing the difference between transcript abundance and protein abundance for three pairs of species and one intraspecific pair. We found no evidence for buffering during divergence of our three species pairs but did find evidence for protein-level buffering for our single intraspecific comparison, suggesting that buffering, if present, was a transient phenomenon in venom divergence. Our results demonstrated that post-transcriptional mechanisms did not contribute significantly to phenotypic evolution in venoms and suggest a more prominent and direct role for *cis*-regulatory evolution in phenotypic variation, particularly for snake venoms.

The relationship between genotype and phenotype is complex, and how this relationship shapes evolutionary patterns depends on how or whether variation can be produced at each step in the phenotypic expression of the genotype. The production of proteins is a primary step along the genotype-to-phenotype pathway, and equilibrium protein levels are determined by rates of transcription, mRNA degradation, translation, and protein degradation (Li *et al.* 2014; Li and Biggin 2015). Some recent work has suggested that mRNA abundances do not accurately predict final protein levels (Foss *et al.* 2007; Diz *et al.* 2012; Khan *et al.* 2013; Casewell *et al.* 2014), which would imply that post-transcriptional regulatory processes, such as those affecting translational rates, contribute significantly to phenotypes. Furthermore, these post-transcriptional processes appear to buffer changes in transcriptional patterns as species diverge. Schrimpf *et al.* (2009) found higher correlation for protein levels between *Caenorhabditis elegans* and *Drosophila melanogaster* than for transcript levels or even transcript *vs.* protein levels within species. Similar results have been found in the divergence of nematode species (Stadler and Fire 2013) and across bacteria, yeast, flies, humans, and rice (Laurent *et al.* 2010). These results suggest that changes in transcriptional patterns often detected in transcriptome-based studies have little or no effect on the phenotypes undergoing study.

Previously detected discrepancies between mRNA and protein levels might reflect methodological or statistical issues (Li and Biggin 2015). A recent study in mammals, which accounted for methodological and technical issues of previous studies, found a much higher correlation between transcript and protein levels (Li *et al.* 2014); transcript levels explained at least 56% of the differences in protein abundance. In contrast to studies showing protein-level buffering, changes in mRNA levels were recently shown to play a dominant role in changes in protein levels during the response of mammalian cells to pathogens (Jovanovic *et al.* 2015). In addition to technical issues, the degree of correlation between transcript and protein abundances was found to be related to protein function in mice (Ghazalpour *et al.* 2011). Disagreements between studies therefore might also reflect differences in the systems or particular tissues being studied.

Snake venoms are a unique system for the study of the relationship between genotypes and phenotypes. Because they are secretions, the genotype–phenotype relationships for venoms are relatively simple, with no complicating developmental processes interposed between the expressed genes and their final products. Because of their evolutionarily critical roles in feeding and defense (Jansa and Voss 2011) and antagonistic coevolutionary interactions with predators and prey (Biardi *et al.* 2005, 2011), they appear to evolve rapidly [although exceptions are known (Margres *et al.* 2015a)] under diversifying selection, and regulatory changes appear to play a major role in their evolution (Margres *et al.* 2015a; Rokyta *et al.* 2015). Venoms are particularly significant in the context of the transcriptome–proteome relationship. Most previous studies of this relationship examined conserved housekeeping genes, which are expected to have protein levels under stabilizing selection. Protein-level buffering and post-transcriptional regulation could actually be detrimental for traits under directional selection. The first study to compare locus-specific venom gland transcriptome abundances to venom proteome abundances showed an approximate correspondence for two species, *Ovophis okinavensis* and *Protobothrops flavoviridis*, of the family Viperidae (Aird *et al.* 2013). For both species, significant positive correlations were detected, and approximately half of the variance was explained. More recently, Casewell *et al.* (2014) claimed to provide evidence from six viperid species for significant contributions of post-transcriptional regulation to venom composition after having failed to find a high correlation between transcript and protein abundances. This study, however, suffered from a number of flaws that render its conclusions questionable. Foremost among these flaws was the use of Sanger sequencing of cDNA libraries to estimate transcript levels, a method that is generally not quantitative (Wang *et al.* 2009). Their proteomic methods (Calvete *et al.* 2007b) were also low-resolution, relying on a convolution of HPLC absorbance values and gel densitometry to estimate protein abundances. In fact, Casewell *et al.* (2014) showed reasonable correspondence between transcript and protein levels when they reduced the resolution to toxin gene families rather than attempting to estimate abundances for individual paralogs. Disagreement between transcriptomes and proteomes could reflect a significant biological phenomenon, but, particularly in cases of disagreement, technical limitations must first be rejected as the source of the pattern.

To determine whether venom gland transcript levels and venom protein abundances were positively correlated and thereby ascertain the role of post-transcriptional regulation in venom evolution, we analyzed venom gland transcriptomes and venom proteomes from 11 snakes from 10 species, representing three families and six genera. These species included six species from the family Viperidae (*Crotalus adamanteus*, *Crotalus horridus*, *Agkistrodon contortrix*, *Agkistrodon piscivorus*, *Sistrurus catenatus*, and *Sistrurus miliarius*), two species from the family Elapidae (*Micrurus fulvius* and *Micrurus tener*), and two species from the family Colubridae (*Boiga irregularis* and *Hypsiglena* sp.). We included two individuals of *C. horridus* representing a known case of rapid intraspecific venom evolution (Glenn *et al.* 1994; Rokyta *et al.* 2015). We tested for protein-level buffering by comparing divergence in transcript *vs.* protein abundances for orthologous toxins across four pairs of snakes to determine the importance of post-transcriptional mechanisms during species divergence.

## Materials and Methods

### Transcriptome sequencing and assembly

The transcriptomes for *B. irregularis* (McGivern *et al.* 2014), *Hypsiglena* sp. (McGivern *et al.* 2014), *C. adamanteus* (Rokyta *et al.* 2011; Margres *et al.* 2015a), and *M. fulvius* (Margres *et al.* 2013) and the two for *C. horridus* (Rokyta *et al.* 2013, 2015) were described previously. Sequencing for *B. irregularis* and *Hypsiglena* sp. was performed on an Illumina MiSeq with 150-nucleotide paired-end reads. All other sequencing was performed on an Illumina HiSeq with 100-nucleotide paired-end reads. Assembly proceeded exactly as described by Rokyta *et al.* (2015). Our specimen of *Hypsiglena* was from an undescribed species (McGivern *et al.* 2014), hence its designation as *Hypsiglena* sp. Mean insert sizes of all libraries were 130–150 nucleotides. Summaries of the sequencing data are provided in Table 1.

**Table 1:** Summary of transcriptome sequencing and assembly

Species	Read Length	No. of Pairs	Read Qual.	Merged Reads	Merged Length	Merged Qual.	No. of Toxins	TSA Accession	SRA Accession
*Crotalus adamanteus*	101	95,643,958	32	60,687,972	143	38	44	GDBB01000000	SRR441163
*Crotalus horridus* A	100	104,457,593	32	61,150,973	135	38	42	GDBC01000000	SRR575168
*Crotalus horridus* B	100	62,494,397	36	42,425,941	133	38	49	GDBD01000000	SRR1554232
*Micrurus fulvius*	101	79,573,048	31	52,624,077	137	38	43	GDBF01000000	SRR630454
*Micrurus tener*	100	57,428,210	35	40,482,723	135	38	59	GDBH01000000	SRR2028245
*Boiga irregularis*	151	17,103,141	35	16,340,720	143	39	46	GDBA01000000	SRR1292619
*Hypsiglena* sp.	151	16,103,579	36	15,858,156	142	39	33	GDBE01000000	SRR1292610
*Sistrurus catenatus*	100	102,409,559	32	60,426,084	137	38	71	GDBI01000000	SRR2029826
*Sistrurus miliarius*	100	114,684,764	33	72,767,908	140	38	63	GDBJ01000000	SRR2031930
*Agkistrodon contortrix*	100	103,979,548	25	31,169,225	136	38	69	GDAY01000000	SRR2032114
*Agkistrodon piscivorus*	101	69,571,375	32	40,523,629	144	38	76	GDAZ01000000	SRR2032118

### Estimating transcript abundances

To estimate transcript abundances for each transcriptome, we first generated sets of merged reads for each data set using PEAR (Table 1) (Zhang *et al.* 2014). Read merging helps eliminate low-quality bases, facilitates the removal of any adapter read-through because of short insert sizes, and generates longer composite reads that are more likely to have a unique mapping. All estimates were made using only these high-quality merged reads. All unique venom transcripts for each transcriptome were clustered into sets showing less than 1% sequence divergence, and only one representative from each cluster was used in our analyses. Members of clusters represent alleles, recently derived paralogs, or contigs incorporating sequencing errors with differences below the resolution afforded by our sequencing read lengths. We estimated transcript abundances by mapping reads to only the coding sequences of toxin-encoding transcripts. We used three different methods for estimating transcript abundances. We used SeqMan NGen version 12.2 with 10 million merged reads and a 95% minimum match percentage, bowtie2 (Langmead and Salzberg 2012) version 2.2.5 using 10 million merged reads, and RSEM (Li and Dewey 2011) with bowtie (Langmead *et al.* 2009) as the aligner and using all merged reads. For bowtie2, we performed both local and end-to-end alignments and found nearly perfect correlations between these values for all data sets (0.99 < ρ < 1.0 and 0.99 < *R* < 1.0, where ρ is Spearman’s rank correlation coefficient and *R* is Pearson’s correlation coefficient). We therefore only presented the results from local alignments. For NGen and bowtie2, we used read counts as our estimates of transcript abundances. We used the estimate of transcripts per million (TPM) from RSEM. To ensure that only transcripts with accurate estimates of abundance were included in our analyses, we eliminated those with coefficients of variation greater than 1 in their coverage across sites on the basis of the bowtie2 local alignments. We also compared the percentage of mapped reads for each transcript from the bowtie2 local alignments and the estimated percentages of read counts per transcript with RSEM. Transcripts with 10-fold or higher differences between methods were excluded.

### Mass spectrometry

Mass spectrometry analysis of venom was conducted by the Florida State University College of Medicine Translational Science Laboratory. To analyze whole venom samples, we performed nanospray LC/MS^E^ using the Synapt G2 HD Mass Spectrometer with a nanoAcquity UPLC (Waters Corp.) MS^E^ is a data-independent acquisition mode that alternates between low and high energy functions, collecting mass spectral data for all detectable precursor and product ions. For coeluting peptides, the high energy spectra are chimeric, containing a mixture of unfragmented precursor ions and CID fragment ions from multiple precursors. Digestion of whole venom samples was performed using the Calbiochem ProteoExtract All-in-One Trypsin Digestion Kit (Merck, Darmstadt, Germany) according to the manufacturer’s instructions using LC/MS grade solvents. Whole venom digests were diluted 1:10 in 3% acetonitrile (ACN) in LC/MS grade water (J. T. Baker) with 0.1% formic acid (FA) and 25 fmol/μl yeast alcohol dehydrogenase (ADH, Waters Corp.) as an internal standard. Two μL of sample containing 400 ng venom and 50 fmol of the internal standard (ADH) was injected. Glufibrinopeptide (785.8426 m/z; Waters Corp.) was used as the lock mass (external calibrant). Tryptic peptides were separated by reverse-phase chromatography using a Waters nanoAcquity UPLC BEH130 C18 column with dimensions of 100 um × 100 mm and 1.7 μm bead size. Gradient conditions were as follows: mobile phase A solvent was 0.1% formic acid (Aq); mobile phase B solvent was 0.1% formic acid in acetonitrile (ACN); column was maintained at a temperature of 35° and a flow rate of 880 nL/min. The column was pre-equilibrated at initial conditions of 7% B and the gradient proceeded 7–35% B over 55 min, 35–50% B over 5 min, 50–80% B over 2 min, and remained at 80% B for 5 min before returning to 7% B over 3 min. Data were acquired for 70 min in nanoESI Positive mode over a mass range of 50–2000 m/z. The ion source temperature was 80°, capillary and cone voltages were 2.8 kV and 30 V, respectively, and nanoflow gas was 0.5 bar. Fragmentation occurred in the trap collision cell with low energy collision set at 4 V and high energy collision set over a ramp of 15–40 V. Raw data were generated using MassLynx version 4.1 software (Waters Corp.) and data were processed in ProteinLynx Global SERVER (PLGS) version 2.5.1 (Waters Corp.). The IdentityE function in PLGS was used to deconvolute the spectra by assigning fragment ions to specific precursors based on retention time and other factors. Proteins were identified using the PLGS IdentityE algorithm to search a decoy database containing entries specific to the proteome animal with the internal standard sequence (ADH) appended and an equal number of reversed sequences. The database included all putative toxin proteins as well as the nontoxin proteins identified in the venom-gland transcriptome. Search parameters allowed for precursor and fragment mass tolerances to be set by the software based on resolution, one missed cleavage site, three peptides per protein, seven fragment ions per protein, and post-translational modifications of cysteine carbamidomethylation (fixed) and oxidation of methionine (variable). Each sample was run in triplicate.

### Estimating protein abundances

All proteins retained in the final analyses had 0% false-positive rates (FPR), and any protein not detected in all three replicates was excluded from our quantitative comparisons. Yeast alcohol dehydrogenase (ADH) was used as an internal standard for calculating response factors in the estimation of protein quantities, and response factors were calculated independently for each replicate. For the first analysis, only the top three peptides, ranked by PLGS score (PLGS version 2.5.1), were used for protein quantification. Only proteins with at least three detected peptides were retained. The known load of ADH (50 fmol) was divided by the summed intensities of its top three peptides to obtain a response factor with units of fmol per unit intensity. The response factor was multiplied by the sum of the top three peptide intensities for each venom protein to estimate its concentration. For the second analysis, we used the summed intensities of all peptides in our calculations. For the response factor, the known concentration of ADH (50 fmol) was multiplied by its molecular weight to obtain the total fg load. This fg value was divided by the summed intensities of all ADH peptides to obtain a response factor with units of fg per unit intensity. The response factor was multiplied by the summed intensities of all peptides in each venom protein to estimate its total fg load, which was then divided by the theoretical molecular weight of the venom protein to estimate its fmol concentration. This all-peptide quantification method is based on the same principles underlying iBAQ, which has been demonstrated to have biological relevance and to perform well at the protein and proteome levels (Schwanhäusser *et al.* 2011; Arike *et al.* 2012). For both analyses, fmol values from each replicate were calculated separately and averaged to produce a final estimate of concentration. To assess the quality of our estimates, we calculated the coefficients of variation for the values of each protein across replicates. From all 11 data sets, only a single protein had a coefficient of variation greater than 1 under either analysis: *C. adamanteus* SVSP-1 = 1.05 under the all-peptide analysis. Across all 11 data sets, the coefficients of variation were below 0.3 for 93% of proteins under the all-peptide analysis and 96% of proteins under the top-three analysis. We therefore did not exclude any proteins on the basis of low quality. We did, however, exclude all Bradykinin-potentiation and C-type natriuretic peptides because they are known to undergo extensive proteolytic cleavage, which could cause a significant discrepancy between the predicted and actual peptides.

### Selecting abundance measures

To compare transcript to protein levels, we needed comparable measures of both. For transcript abundances, we began with counts of reads mapped to coding sequences from alignments using NGen version 12 from the DNASTAR software suite (DNASTAR, Inc., Madison, WI) and bowtie2 (Langmead and Salzberg 2012) and transcripts per million (TPM) estimated using a bowtie (Langmead *et al.* 2009) alignment and RSEM (Li and Dewey 2011). All of these measures were highly correlated across all 11 data sets (NGen *vs.* bowtie2: 0.99 < ρ < 1.0 and 0.99 < *R* < 1.0, NGen *vs.* TPM: 0.87 < ρ < 0.98 and 0.74 < *R* < 0.98, and bowtie2 *vs.* TPM: 0.87 < ρ < 0.98 and 0.74 < *R* < 0.98, where ρ is Spearman’s rank correlation coefficient and *R* is Pearson’s correlation coefficient). For protein abundances, we estimated molar amounts using two approaches. For the first, we only considered the best three peptide matches for each protein. For the second, we used all identified peptides. These measures of protein abundance were also highly correlated across all 11 data sets (0.84 < ρ < 0.97; 0.68 < *R* < 0.99). For our comparisons, we therefore used TPM estimates for transcript abundances, because these are most directly analogous to molar amounts, and the all-peptide protein abundances, because this measure should be less sensitive to shared peptides among paralogs in the large gene families characteristic of snake venoms.

### Testing for post-transcriptional silencing

To test for the presence of venom-encoding transcripts expressed at high levels but not detectable proteomically, we first excluded from consideration transcripts with anomalous coverage distributions or high discrepancies between transcript estimates as well as all Bradykinin-potentiation and C-type natriuretic peptides for the reasons described above. A transcript was considered proteomically detected if it was found in at least one of the three mass spectrometry replicates with a 0% FPR.

### Statistical analyses

All statistical analyses were conducted in R (R Development Core Team 2006). For all of our transcript-level and protein-level comparisons, we used a centered log-ratio (clr) transform on the raw abundance estimates after normalizing them to sum to 1 (Aitchison 1986). If the normalized data are $x=\left(x_{1},\ldots ,x_{n}\right)$ such that $\sum_{i=1}^{n}x_{i}=1$, then

$$\text{clr}\left(x\right)=\left(\ln\frac{x_{1}}{g\left(x\right)},\ldots ,\ln\frac{x_{n}}{g\left(x\right)}\right)$$ (1)

where $g\left(x\right)=\sqrt[{n}]{x_{1}\cdots x_{n}}$ is the geometric mean. This transformation takes the data from the simplex to real space. Because this transform preserves rank, Spearman’s rank correlation coefficients were unaffected. Because g(x) is the same for each component in each data set and log(x/y)=logx−logy, the clr transform merely shifts all of the points in a data set by a constant amount relative to the standard log transform. For linear relationships, the clr transformation is therefore equivalent to a log transform.

The choice of the clr transformation was made on the basis of theory (Aitchison 1986) related to the treatment of data that are sum-constrained. Although not widely recognized (but see Vêncio *et al.* 2007; Rokyta *et al.* 2015), RNA-seq data suffer from this constraint, because the number of reads generated is independent of what is being sequenced. Proteomic data suffer from the same issue (Margres *et al.* 2015a). This can be understood most clearly by noting that for neither approach do we have a meaningful way of measuring biologically relevant absolute quantities. The use of log-ratio transforms reflects an acknowledgment that we can only meaningfully compare relative quantities of our components (*i.e.*, transcripts or proteins).

### Data availability

All raw transcriptomic reads were deposited in the National Center for Biotechnology Information (NCBI) Short Read Archive (SRA), and the assembled toxin transcripts were deposited in the NCBI Transcriptome Shotgun Assembly (TSA) databases. Accession numbers are provided in Table 1. The mass spectrometry proteomics data have been deposited to the ProteomeXchange Consortium (Vizcaíno *et al.* 2014) via the PRIDE partner repository with the dataset identifier PXD002837.

## Results and Discussion

### Minimal contribution of post-transcriptional regulation to protein abundances

**Figure 1:**
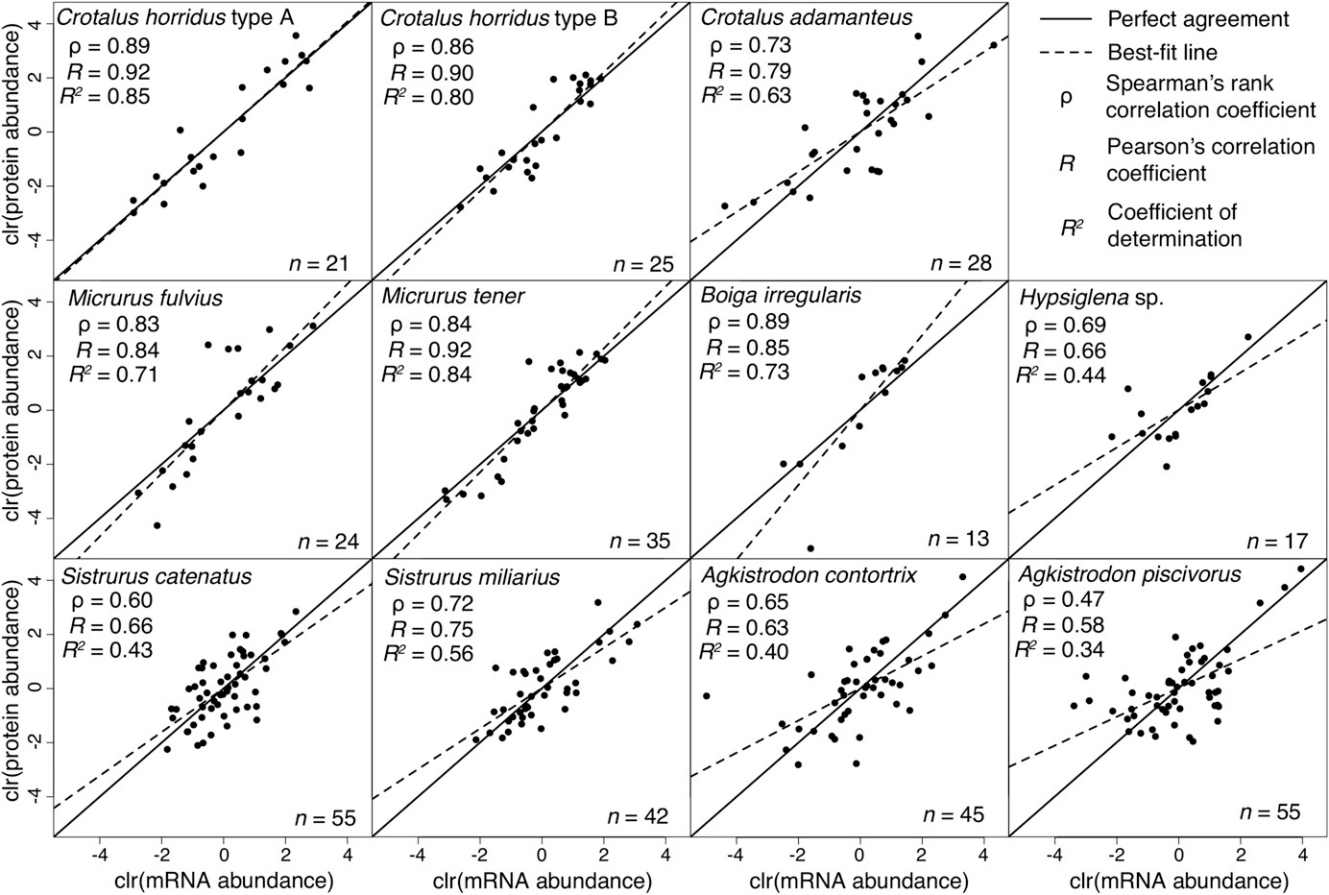
Protein and mRNA abundances were highly correlated between venom proteomes and venom gland transcriptomes. We compared RSEM estimates of transcripts per million (TPM) from venom gland transcriptomes to molar estimates of protein abundances in venoms for 11 individual snakes from 10 species and three families. All data were centered log-ratio (clr) transformed. Spearman’s rank correlations were high (ρ > 0.6) for 10 of 11 comparisons. Transcript abundances explained the majority of variation in protein abundance (*i.e.*, *R*^2^ > 0.5) for seven of the 11 comparisons.

Transcript and protein abundances were highly correlated across three snake families. Although the strength of correlation varied among comparisons (0.47 < ρ < 0.89, where ρ is Spearman’s rank correlation coefficient), 10 of the 11 comparisons showed ρ>0.6, indicating that the prevailing pattern was a strong agreement between transcript and protein abundances for venoms and venom glands (Figure 1). Five comparisons (*B. irregularis*, *C. horridus* type A, *C. horridus* type B, *M. fulvius*, and *M. tener*) showed ρ > 0.8, clearly indicating that the transcriptome can accurately predict the proteome. We also found a strong linear relationship between transcript and protein levels (Figure 1). Pearson’s correlation coefficients (*R*) ranged from 0.58 to 0.92, with five of 11 comparisons giving *R* > 0.8. Transcript abundances explained the majority of variation in protein abundance (*i.e.*, *R*^2^ > 0.5) in seven of the 11 comparisons. This high level of agreement held across three families and varying levels of venom complexity (Figure 1).

Our results clearly demonstrated a strong agreement between transcript and protein levels for venom glands and venom. We identified cases of higher levels of transcriptome/proteome concordance than ever previously reported (*e.g.*, ρ = 0.89, *R* = 0.92, and *R*^2^ = 0.85 for *C. horridus* type A) and showed similar patterns of agreement across three snake families. This agreement is remarkable given that we were comparing equilibrium protein levels to nonequilibrium transcriptional levels; our transcriptomes characterized one time point during the whole process of venom production, suggesting little temporal heterogeneity in transcriptional levels among venom transcripts during venom production. Although some variance in protein levels remains to be explained, the invocation of “post-genomic mechanisms” as major contributors to venom composition variation (Casewell *et al.* 2014) appears to have been premature. The transcriptome can be a strong predictor of the proteome.

### No protein-level buffering during species divergence

Selection acts on protein rather than transcript levels (Diz *et al.* 2012) and, under most conditions, protein levels are under stabilizing selection and stronger constraints than transcript levels (Khan *et al.* 2013). Previous work (Schrimpf *et al.* 2009; Laurent *et al.* 2010; Stadler and Fire 2013) has shown that divergence in transcript abundances between species is buffered at the protein level; the efficacy of stabilizing selection is thereby enhanced through post-transcriptional regulatory processes. Because of venom’s central role in the evolution and ecology of venomous snakes (Fry and Wüster 2004), venom composition evolves quickly under diversifying selection as species (Calvete *et al.* 2007a, 2010) or populations (Alape-Girón *et al.* 2008; Gibbs *et al.* 2009; Núñez *et al.* 2009; Boldrini-França *et al.* 2010; Calvete *et al.* 2011; Margres *et al.* 2015a; Rokyta *et al.* 2015) diverge. Under diversifying selection, protein-level buffering could hinder adaptation. We therefore tested whether the variation in protein levels unexplained by transcript levels (Figure 1) was biased toward maintaining similarity in protein abundances as has been previously described for proteins more likely under stabilizing selection.

**Figure 2:**
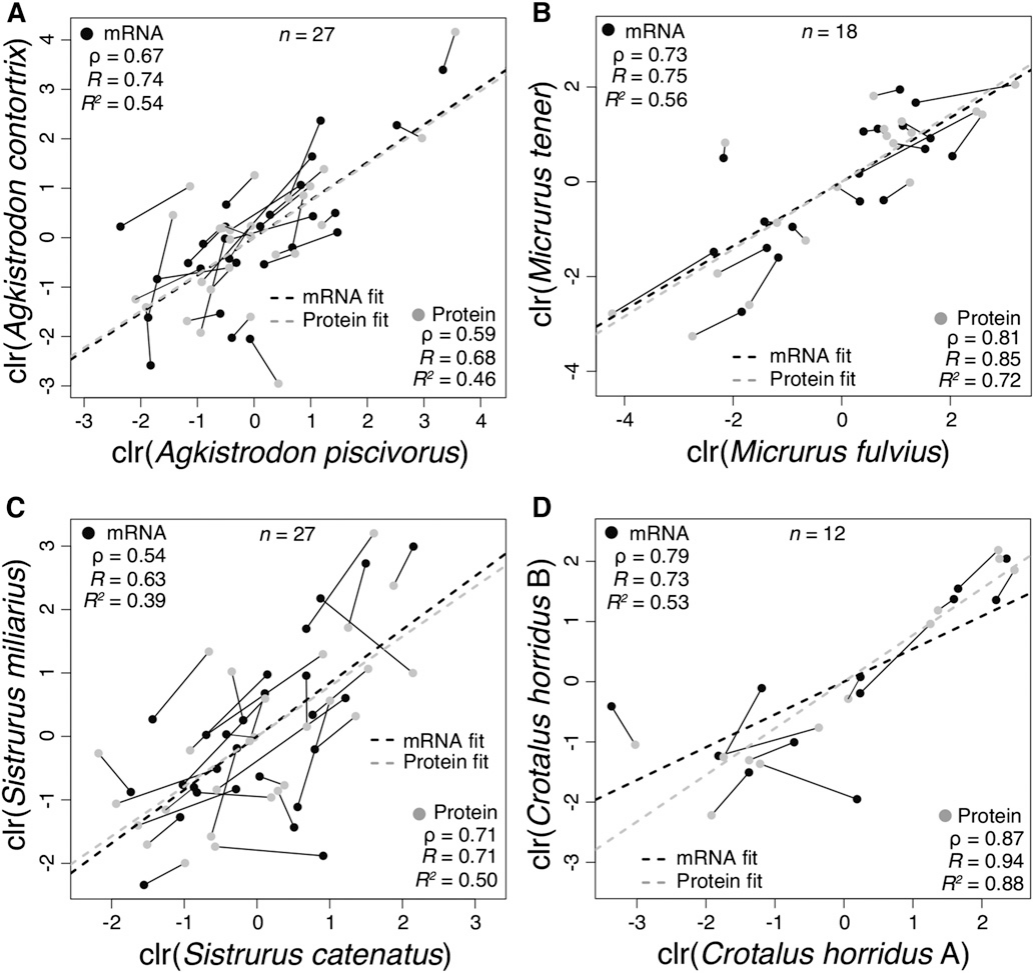
Protein-level expression buffering was not observed in three interspecific comparisons of pairwise divergence in venom composition but was observed for intraspecific divergence. Orthologous proteins were identified by means of reciprocal blastp searches. Transcript values are shown in black, and protein values are shown in gray. Values for corresponding transcript/protein pairs are connected by line segments. In the three interspecific comparisons, protein divergence between pairs was statistically indistinguishable from transcript divergence, indicating that divergence could be accounted for by changes in transcript levels alone. In the intraspecific comparison for *Crotalus horridus*, protein-level divergence was less than transcript-level divergence, indicating the presence of buffering.

We considered expression evolution in three pairs of recently (less than 10 million years) (Guiher and Burbrink 2008; Kubatko *et al.* 2011; Castoe *et al.* 2012) diverged species: *A. piscivorus* and *A. contortrix*, *M. fulvius* and *M. tener*, and *S. miliarius* and *S. catenatus*. We found indistinguishable correlations between protein levels across species and transcript levels (Figure 2). For the *Agkistrodon* pair (Figure 2A), we found *R* = 0.74 with a 95% confidence interval (CI) of (0.50, 0.87) for transcript abundances and *R* = 0.68 with a 95% CI of (0.40, 0.84) for protein abundances. For the *Micrurus* pair (Figure 2B), we found *R* = 0.75 with a 95% C.I. of (0.43,0.90) for transcript levels and *R* = 0.85 with a 95% CI of (0.63, 0.94) for protein levels. For the *Sistrurus* pair (Figure 2C), we found *R* = 0.63 with a 95% CI of (0.32,0.81) for transcripts and *R* = 0.71 with a 95% CI of (0.45, 0.86) for proteins. In all three cases, the estimates of *R* for protein levels were well within the CIs of transcript levels, and the estimates of *R* for the transcript levels were well within the CIs of the protein levels. We therefore found that divergence in venom composition can be explained by changes in transcriptional patterns and found no evidence at the species level for protein-level buffering for a trait under diversifying selection.

We found evidence for protein-level buffering for our single intraspecific comparison (Figure 2D). Comparing *C. horridus* with type A (neurotoxic) and type B (hemorrhagic) venoms, we found *R* = 0.73 with a 95% C.I. of (0.27, 0.92) for transcript abundances and *R* = 0.94 with a 95% CI of (0.79, 0.98) for protein abundances. Each estimate of *R* was outside the CI associated with its counterpart, suggesting a significant difference in the extent of divergence between protein quantities and transcript quantities. Protein levels were more highly correlated than transcript levels, indicating that changes in transcriptional patterns were buffered post-transcriptionally. Much of the difference between these two venom types involved loss of transcription of venom genes (*i.e.*, was due to pretranscriptional regulation) in the type A venom gland (Rokyta *et al.* 2015), but our analysis could only include toxins detected proteomically. Nonetheless, this apparent protein-level buffering for only our most recently diverged pair of taxa could indicate the initial presence of buffering that, in the case of snake venoms under diversifying selection, is ultimately erased by selection during species divergence.

Protein-level buffering against changes in transcriptional patterns appeared to be, at best, a transient phenomenon during species divergence. Three of our four comparisons clearly showed that divergence, presumably under directional selection, of venom composition was affected through changes in transcriptional patterns and that these changes are quantitatively reflected in the venom proteomes. Because venoms are secretions, protein degradation is eliminated as a potential post-transcriptional mechanism for buffering, thereby limiting the number of mechanisms available to accomplish buffering. In addition, ontogenetic changes in snake venom composition are widespread (Minton 1975; Mackessy 1988, 1993; Andrade and Abe 1999; López-Lozano *et al.* 2002; Lamar 2003; Mackessy *et al.* 2003; Guércio *et al.* 2006; Wray *et al.* 2015; Margres *et al.* 2015b), indicating that rapid expression-mediated changes are necessary in venom-gland tissue. Buffering would hinder these changes as well as the response to directional selection and may therefore be inactive in these tissues, if such mechanisms exist at all.

### The role of post-transcriptional silencing

**Figure 3:**
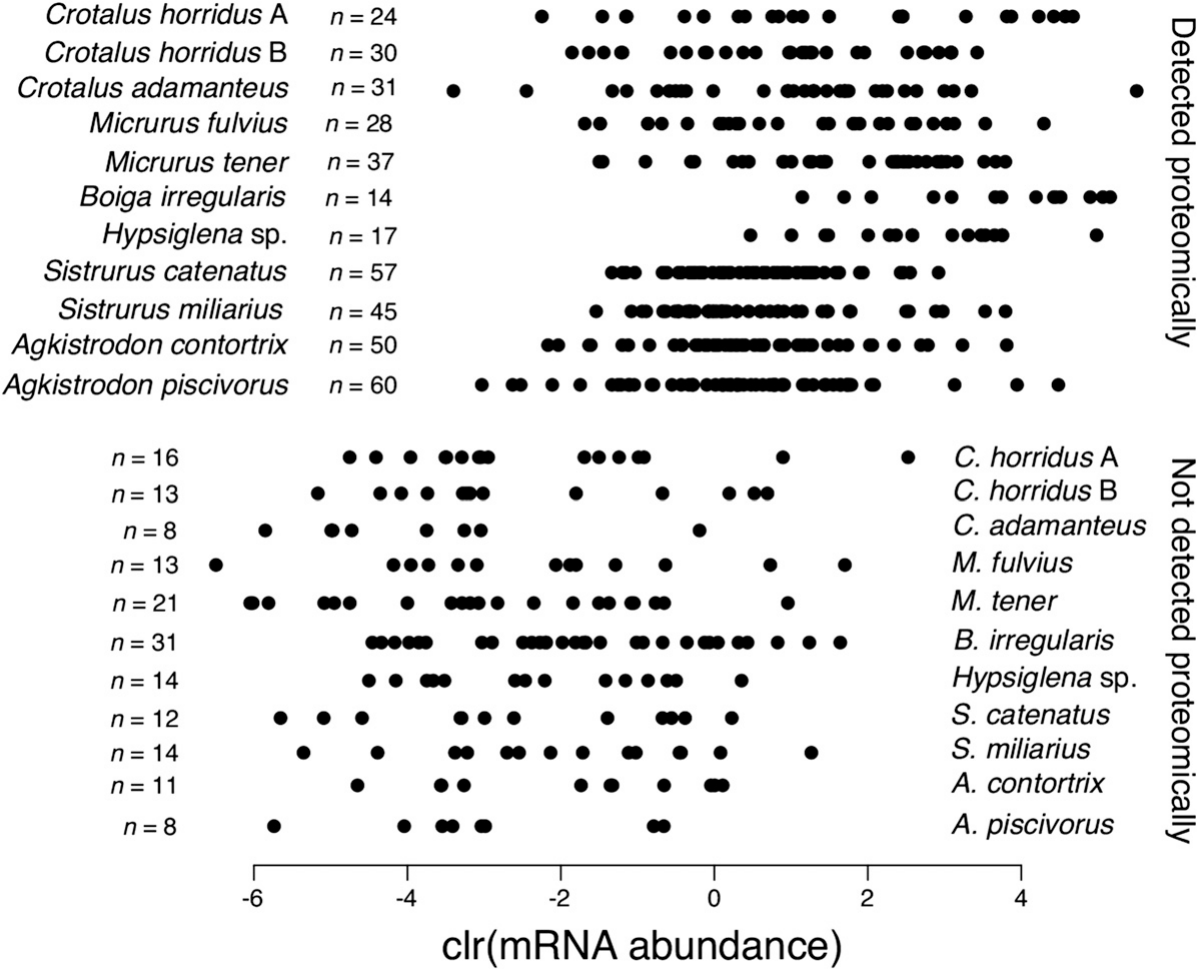
Nearly all highly expressed putative toxins transcripts were detected proteomically. Transcripts were identified as putative toxins on the basis of homology with known toxins. Failure to detect these putative toxins proteomically could reflect post-transcriptional silencing, misassignment as toxins, or simply a proteomic detection threshold. The undetected putative toxin transcripts were nearly all expressed at relatively low levels, suggesting a detection threshold. We found no evidence for highly expressed putative toxins being post-transcriptionally silenced.

We showed a strong quantitative agreement between venom gland transcriptomes and venom proteomes (Figure 1), but these analyses ignored, by necessity, the possibility of qualitative disagreements. Such qualitative disagreements, particularly the failure to detect the protein products of highly expressed putative toxin transcripts, have been used to argue for a role of post-transcriptional regulation or temporally varying expression patterns in venom glands (Calvete *et al.* 2007c; Sanz *et al.* 2008; Wagstaff *et al.* 2009). Concordance between transcriptomes and proteomes (qualitative or quantitative) is unlikely to result from biases or technical limitations. Discordance, however, can arise through true biological phenomena or through methodological issues; the burden of proof for establishing discordance is more substantial. Failure to detect a predicted protein could reflect a detection threshold for our proteomic approach, erroneous classification of a transcript as toxin-encoding, or some form of post-transcriptional silencing. Loss of expression of toxin transcripts during evolution could also be leaky, showing low levels of residual transcription, but this phenomenon could also be considered to result from a proteomic detection threshold. A detection threshold would bias analyses toward detecting the proteins encoded by more abundant transcripts. Post-transcriptional silencing and toxin misidentification would presumably affect transcripts at all expression levels. For our 11 transcriptome/proteome comparisons, we found a strong bias against detecting proteins corresponding to the transcripts with the lowest expression levels (Figure 3), consistent with a simple proteomic detection threshold. All of the most highly expressed putative toxin transcripts were detected proteomically, indicating that post-transcriptional silencing of highly expressed toxins was not a major driver of phenotypic evolution for traits evolving under diversifying selection.

### Conclusions

The production of proteins is a major link in the genotype–phenotype relationship, and post-transcriptional regulation has recently been implicated as a significant source of phenotypic variation for a broad array of species. Examinations of the roles of post-transcriptional regulation have focused on proteins with abundances that are most likely under stabilizing selection. We established an extremely simple genotype–phenotype relationship for snake venoms, a trait commonly under diversifying selection, across three major venomous families (Colubridae, Viperidae, and Elapidae) by showing that most variation in protein abundances can be explained without invoking post-transcriptional regulation. We also showed a lack of protein-level buffering during species divergence, except for perhaps during the early stages, and failed to find evidence for post-transcriptional silencing. Altogether, our results showed that changes to the transcriptome drive the evolution of snake venom composition.
